# Sorting trash from treasure: Separate pathways for autophagy and endocytic trafficking in axons

**DOI:** 10.1080/27694127.2023.2166322

**Published:** 2023-01-19

**Authors:** Vineet Vinay Kulkarni, Sandra Maday

**Affiliations:** Department of Neuroscience, Perelman School of Medicine at the University of Pennsylvania, Philadelphia, PA, USA

**Keywords:** α-synuclein, autophagosome, axon, endosome, lysosome, neuron

## Abstract

Macroautophagy (hereafter autophagy) and endocytic trafficking are key pathways in neuronal axons that regulate the composition and integrity of the axonal proteome. These pathways have similar trafficking itineraries; however, the extent of their cross-talk remains incompletely understood. Our recent work demonstrates that under physiological conditions, axonal autophagy and endocytic pathways are separate and exhibit distinct rates of organelle maturation. Strikingly, overexpression of pathogenic α-synuclein disrupts the segregation between these pathways by merging autophagosomes and endosomes generated in the distal axon. These results raise the possibility that precocious degradation of endocytosed cargo via misrouting into lysosome-destined autophagosomes may contribute to neuronal dysfunction in Parkinson disease and related α-synucleinopathies.

The complex morphology of neurons necessitates efficient mechanisms for the separation of cargo molecules that are targeted for lysosomal degradation from those that are essential for survival signaling. Neurons encounter this sorting challenge at the distal end of their axons, which is a primary site for the initiation of autophagy and endocytic trafficking, two key pathways that regulate the axonal proteome. These pathways share many features; autophagosomes and endosomes form in the distal axon and travel vectorially across a vast spatial landscape to reach the soma where they can feed into degradative lysosomes. The degree of overlap between these autophagic and endocytic pathways, however, is poorly understood. Endosomes carry diverse cargos that are destined for lysosomal degradation or evade lysosomal degradation to facilitate signaling processes. Autophagy is canonically viewed as a degradation pathway; however, recent evidence suggests that autophagosomes may merge with signaling endosomes to convey NTF (neurotrophin)-mediated information to the soma. Because dysfunction in these pathways, including the premature degradation of essential signaling information, can cause neurodevelopmental or neurodegenerative disorders, it is critical to elucidate the dynamics, molecular details, and extent of cross-talk between autophagy and endocytic trafficking in axons. In other words, how do neurons sort “garbage from gold”?

In our recent study [1], we examined the extent of overlap between newly-formed endosomes and autophagosomes in axons using live-cell confocal microscopy. To spatially separate axons from the somatodendritic region, we cultured primary neurons in compartmentalized microfluidic devices. In this system, only axons can cross through the micro-channels to reach a distal chamber that is segregated from their corresponding somas. To track newly-formed endosomes, we applied two different types of fluorescently-labeled cargos to the chamber enriched for distal axons: (1) BSA tagged with Alexa dyes to non-specifically label bulk endosomes, and (2) BDNF conjugated to Quantum Dots to label a subpopulation of endosomes termed signaling endosomes. We then performed live-cell imaging in mid-axons. Strikingly, we found that axonal autophagosomes (labeled with GFP-LC3) do not overlap or co-migrate with newly-formed endosomes. Thus, we find that retrograde pathways for autophagy and endocytic trafficking in axons are parallel and separate (Fig. 1A, B).

**Figure 1.**
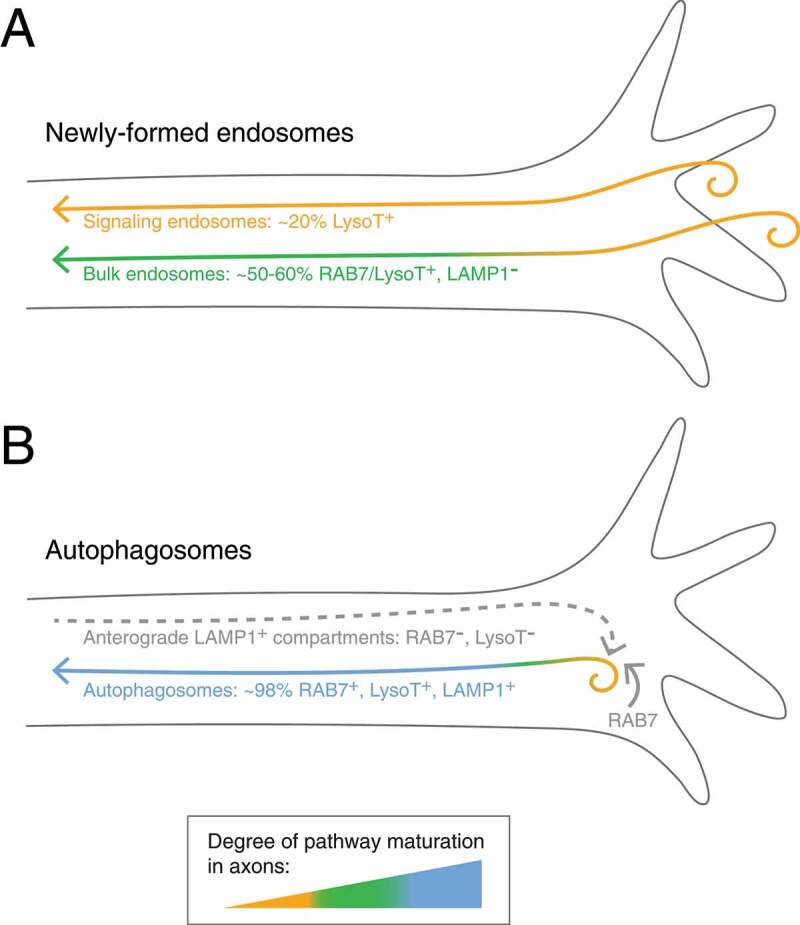
Molecular details of parallel pathways for endosomes and autophagosomes generated in distal axons. (**A**) Newly-formed endosomes labeled in bulk are heterogenous in their maturation state, likely reflecting the diversity of cargos transported in distinct subpopulations of endosomes. For example, signaling endosomes represent a subpopulation of endosomes that are largely not acidic to preserve the integrity of essential signaling-receptor complexes. (**B**) Running parallel and separate to newly-formed endosomes, autophagosomes are also formed in the distal end of the axon and quickly mature into acidic organelles positive for RAB7 and LAMP1. Our observations that autophagosomes do not merge with newly-formed endosomes (which are largely LAMP1 negative), supports observations that the LAMP1 on autophagosomes is derived from anterograde carriers. Anterograde LAMP1 carriers are not acidic and are largely negative for RAB7, suggesting these organelles represent post-Golgi carriers that deliver lysosomal proteases to autophagosomes to confer some degree of proteolytic activity. RAB7 is likely acquired distally. LysoT; LysoTracker.

Next, we wondered whether axonal autophagosomes and newly-formed endosomes differ in their maturation state. Maturation into degradation-competent organelles requires acidification to activate proteases. Thus, we first measured the degree of acidification of axonal organelles in either pathway using LysoTracker, a cell-permeant dye that accumulates in acidic compartments of pH <~6. We found that nearly all axonal autophagosomes are acidic in nature. By contrast, newly-formed endosomes are heterogenous in their degree of acidity, and only a small population (~20%) of BDNF-positive signaling endosomes are acidic. These results suggest that autophagosomes are further along the maturation spectrum, as compared to newly-formed endosomes in axons, which may serve to initiate breakdown or denaturation of cargos (Fig. 1B). The heterogeneity in newly-formed endosomes may reflect the diversity of cargos transported (Fig. 1A). Consistent with this point, the lack of acidity of signaling endosomes likely preserves active NTF-receptor complexes (e.g. BDNF-NTRK2/TrkB) to enable their signaling functions upon arrival in the soma.

To further explore differences in maturation states, we assessed molecular signatures of organelle maturation, namely the late endosome/lysosome-associated protein LAMP1 and the late endosomal GTPase RAB7. Previous work from our lab and others has demonstrated that nearly all axonal autophagosomes are positive for LAMP1 and RAB7 (Fig. 1B). In our current study, we found that only ~60% of newly-formed endosomes are positive for RAB7, and most are negative for LAMP1 (Fig. 1A). Our results indicate that distally-formed autophagosomes and endosomes travel the axon in separate organelle populations that are distinguished in their maturation states (Fig. 1A, B). These findings suggest that endocytosed cargo are sorted in the distal axon before endosomes embark on their long journey to the soma. Interestingly, we detected little proteolytic degradation in newly-formed endosomes until they reached the soma, suggesting additional sorting mechanisms to be decoded locally within the soma. By contrast, the more mature state of autophagosomes is consistent with other studies, which suggest that axonal autophagosomes may possess some degree of proteolytic capacity. In sum, the axon exhibits distinct trafficking itineraries for autophagosomes and endosomes to separate trash from treasure.

Last, we determined whether separation of axonal autophagosomes and newly-formed endosomes is disrupted under pathological conditions. Hence, we overexpressed α-synuclein in neurons and assessed the overlap between autophagic and endocytic pathways in axons. Elevated expression of wild-type (WT) or the A53T mutant of α-synuclein has been causally linked to Parkinson disease. α-synuclein localizes predominantly to distal axons and is proposed to regulate vesicle trafficking. We found that overexpression of WT or A53T α-synuclein increases the presence of newly-endocytosed cargo in retrograde autophagosomes in axons. Thus, elevated expression of WT or A53T α-synuclein may induce merging of axonal autophagosomes and newly-formed endosomes, possibly by altering organelle tethering, fusion, or both. Interestingly, the severity of these effects corresponds to α-synuclein pathogenicity, i.e., A53T α-synuclein increases overlap more than WT α-synuclein, which is higher than controls. We propose that this merging may result in the precocious degradation of endocytosed cargos that should be preserved. Notably, though the increase in merging of autophagy and endocytic pathways is mild, the effects of misrouting essential signaling information may accumulate over the extended life of the neuron and contribute to neuronal dysfunction in α-synucleinopathies.

In contrast to axons, our previous work demonstrates significant merging between autophagy and endocytic pathways in dendrites. These differences may reflect compartment-specific demands for local degradation in remodeling the postsynaptic proteome in response to synaptic activity. Newly-formed endosomes in dendrites may contain a higher proportion of cargo destined for degradation, leading to their increased merging with autophagic organelles. Further, degradation products of autophagy can fuel new protein synthesis, which is a critical component in postsynaptic compartments to facilitate learning and memory.

Our work highlights the compartment-specific organization, function, and cross-talk between autophagy and endocytic trafficking in neurons. We suggest that aberrant merging of axonal autophagy and endocytic pathways may contribute to neuronal dysfunction in Parkinson disease and related α-synucleinopathies. Future studies should determine the identity of the endocytic cargos that are misrouted to autophagosomes in the context of α-synuclein. It will be important to examine the organization of autophagy and endocytic pathways in axons in the presence of exogenous α-synuclein fibrils of synthetic or patient-derived origin. These studies will elucidate the effects of intracellularly- and extracellularly-derived pathogenic proteins on the cross-talk between proteostatic pathways to gain insights into neuronal dysfunction underlying α-synucleinopathies.
